# Rifabutin: a repurposed antibiotic with high potential against planktonic and biofilm staphylococcal clinical isolates

**DOI:** 10.3389/fmicb.2024.1475124

**Published:** 2024-10-10

**Authors:** Magda Ferreira, Margarida Pinto, Frederico Aires-da-Silva, Ana Bettencourt, Maria Manuela Gaspar, Sandra Isabel Aguiar

**Affiliations:** ^1^Center for Interdisciplinary Research in Animal Health (CIISA), Faculty of Veterinary Medicine, Universidade de Lisboa, Lisbon, Portugal; ^2^Associate Laboratory for Animal and Veterinary Sciences (AL4AnimalS), Lisbon, Portugal; ^3^Faculty of Pharmacy, Research Institute for Medicines (iMed.ULisboa), Universidade de Lisboa, Lisbon, Portugal; ^4^Laboratório de Microbiologia do Serviço de Patologia Clínica do Centro Hospitalar Universitário de Lisboa Central, Lisbon, Portugal; ^5^Faculty of Sciences, Institute of Biophysics and Biomedical Engineering (IBEB), Universidade de Lisboa, Lisbon, Portugal

**Keywords:** *Staphylococcus aureus*, antibiotic repurposing, rifabutin, clinical isolates, biofilms

## Abstract

*Staphylococcus aureus* poses a significant threat as an opportunistic pathogen in humans, and animal medicine, particularly in the context of hospital-acquired infections (HAIs). Effective treatment is a significant challenge, contributing substantially to the global health burden. While antibiotic therapy remains the primary approach for staphylococcal infections, its efficacy is often compromised by the emergence of resistant strains and biofilm formation. The anticipated solution is the discovery and development of new antibacterial agents. However, this is a time consuming and expensive process with limited success rates. One potential alternative for addressing this challenge is the repurposing of existing antibiotics. This study investigated the potential of rifabutin (RFB) as a repurposed antibiotic for treating *S. aureus* infections. The minimum inhibitory concentration (MIC) of rifabutin was assessed by the broth microdilution method, in parallel to vancomycin, against 114 clinical isolates in planktonic form. The minimum biofilm inhibitory concentration (MBIC_50_) was determined by an adaptation of the broth microdilution method, followed by MTT assay, against a subset of selected 40 clinical isolates organized in biofilms. The study demonstrated that RFB MIC ranged from 0.002 to 6.250 μg/mL with a MIC_50_ of 0.013 μg/mL. RFB also demonstrated high anti-biofilm activity in the subset of 40 clinical isolates, with confirmed biofilm formation, with no significant MBIC_50_ differences observed between the MSSA and MRSA strains, in contrast to that observed for the VAN. These results highlight the promising efficacy of RFB against staphylococcal clinical isolates with different resistance patterns, whether in planktonic and biofilm forms.

## 1 Introduction

Staphylococci species are the leading cause of healthcare-acquired infections (HAIs), mainly associated with medical device implantation (Tong et al., 2015; Ferreira et al., 2021a,c; Lisowska-Łysiak et al., 2021; Tuon et al., 2023). Among these, *Staphylococcus aureus* is one of the most frequently isolated microorganism in the context of HAIs. *Staphylococcus aureus*, is a gram-positive commensal bacterium that colonizes asymptomatically in over 30% of the human population. This colonization raises the risk of invasive infections, such as bloodstream or internal tissue penetration, especially in instances of compromised host immune systems or surgical interventions (Laux et al., 2019; Taylor and Unakal, 2023). This bacterium is responsible for diseases such as bloodstream infections, endocarditis, osteomyelitis, skin and soft tissue, pleuropulmonary, and device-related infections (Cassat et al., 2014; Ferreira et al., 2021a).

Antibiotics remain the main tool to control infectious diseases, however, their efficacy has been compromised over time due to extensive overuse in both human and animal populations. This has accelerated the emergence of antimicrobial resistance (AMR) (Shajari et al., 2017; Ferreira et al., 2021b; Liu et al., 2021), recognized as a pressing global health issue, as highlighted by the World Health Organization (WHO). This underscores the urgency to explore new alternative treatments.

In this context, managing *S. aureus* infections presents a tremendous challenge, primarily due to the organism’s rapid acquisition of resistance to multiple antibiotic classes. Of particular concern is the widespread prevalence of methicillin-resistant *S. aureus* strains (MRSA) across hospitals, communities, and veterinary environments, posing a significant healthcare menace (Shoaib et al., 2023). *Staphylococcus aureus* treatment is further complicated by its notable ability to form biofilms (Cassat et al., 2014; Idrees et al., 2021). A biofilm is a complex microbial community characterized by cells adhering to either biological or non-biological surfaces and enclosed within a self-produced matrix of extracellular polymeric substances (EPS). These EPS consist of a heterogeneous mixture of polysaccharides, proteins, nucleic acids, and lipids, serving as the foundational scaffold for biofilms and playing crucial roles in their formation, stability, and functionality. *Staphylococcus aureus* may colonize medical devices during the implantation procedures, or even during an asymptomatic or symptomatic bacteremia episodes. Subsequently, the bacteria adhere to the human matrix proteins, such as fibronectin and fibrinogen, thereby facilitating bacterial attachment and ultimately culminating in biofilm establishment (Donlan, 2002; Hall-Stoodley et al., 2004; Flemming et al., 2007; Flemming and Wingender, 2010). Biofilms provide an excellent defense mechanism for bacteria, functioning as a protective barrier from the host immune system and hampering antibiotic penetration into their structure (Ferreira et al., 2021a; Idrees et al., 2021; Tuon et al., 2023). Moreover, bacterial cells present within the biofilms may enter a low metabolic state, which radically increases their tolerance to antibiotics. Thus, eradication of biofilm-related infections requires administration of high doses of antibiotics, which may lead to severe side effects in patients. Given these challenges, the primary therapeutic strategy often involves surgical removing of the established biofilm, an invasive approach not always clinically viable (Idrees et al., 2021; Tuon et al., 2023).

Therefore, effective treatment of biofilm-related infections requires the use of antibiotics with high biofilm penetration rates. Among the available options, tetracyclines, macrolides, lincosamides, quinolones, oxazolidinones, sulfonamides, nitroimidazole, fusidic acid, and rifamycins offer more advantages comparatively to aminoglycosides, polymyxis, β-lactamases, and glycopeptides (Tuon et al., 2023). Indeed, studies have revealed that vancomycin (VAN), a commonly used glycopeptide for treating *S. aureus* biofilm-related infections, particularly those caused by MRSA, exhibits diminished biofilm penetration rates (Jefferson et al., 2005; Singh et al., 2010). Additionally, a considerable reduction in the biofilm penetration rates of β-lactam antibiotics, such as oxacillin and cefotaxime, has been reported (Singh et al., 2010). This highly contributes to prolonged, persistent, or recurrent bacteremia during therapy, leading to high rates of clinical failures, nephrotoxicity and the emergence of non-susceptible strains (Tuon et al., 2023). While rifampicin, a rifamycin antibiotic, is commonly employed in the treatment of *S. aureus* infections, it is associated with a high rate of resistance emergence and therapeutic failure when used as monotherapy. Hence, it is frequently administrated in combination with other antibiotics, such as VAN. Nevertheless, rifampicin is known for its propensity for drug interactions and its potential for adverse side effects (Scholar, 2007; Chambers, 2020).

Considering these limitations, significant attention is being given to the discovery of new antimicrobial agents, such as anti-virulence agents, antibodies, probiotics, and vaccines. While these agents hold promise, they are likely most advantageous as adjunctive or preventive therapies since they have not yet provided sufficient clinical benefit to replace antibiotics (Czaplewski et al., 2016; Liu et al., 2021). The most obvious approach appears to be the discovery and development of new antibiotics. However this is a long process, with high scientific and economic challenges, and potentially hindered by the emergence of antimicrobial resistance as a result of bacterial adaptation (Ferreira et al., 2021c; Liu et al., 2021). Another attractive approach that has been explored is antimicrobial repurposing. The use of approved antimicrobial agents, such as antibiotics for applications outside the scope of the original medical indication, has been described as a favorable strategy to eradicate infections caused by strains resistant to conventional antibiotics (Jing et al., 2022). This approach can potentially bypass the lengthy and costly process of discovery and development of new drugs (Ferreira et al., 2021c; Liu et al., 2021).

In the realm of repurposing antibiotics, alternative rifamycins such as rifabutin (RFB), have garnered attention as potential substitutes for rifampicin (Albano et al., 2019; Chambers, 2020; Doub et al., 2020; Monk et al., 2022; Thill et al., 2022). RFB is a spiro-piperidyl-rifamycin, structurally similar to rifampicin, and demonstrates comparable antibacterial activity. Notably, RFB exhibits a longer half-life, improved tissue penetration, and a reduced incidence of adverse side effects and drug–drug interactions compared to rifampicin (Aronson, 2016; Thill et al., 2022). Although RFB is a broad-spectrum antibiotic with demonstrated antibacterial activity against both Gram-negative and Gram-positive bacteria, clinical indications have been restricted for the treatment of *Mycobacterium tuberculosis* and non-tuberculous mycobacterium (NTM) infections (Gaspar et al., 2008a,b; Crabol et al., 2016).

With the expiration of its patent protection, the price of RFB has been reduced by 60%, leading to an increase of published studies highlighting its promising potential against several bacterial infections (Crabol et al., 2016). Recent studies have revealed a high rate of success in the treatment of *Helicobacter pylori* infection (Gisbert, 2021). Furthermore, in many other studies, the potentiality of RFB in the eradication of *Acinetobacter baumannii*, *Escherichia coli*, *Klebsiella pneumoniae*, *Pseudomonas aeruginosa*, and *S. aureus* clinical isolates has been demonstrated (Albano et al., 2019; Karau et al., 2020; Lee et al., 2021; Thill et al., 2022). Our prior study (Ferreira et al., 2021c), further demonstrated a potent antibacterial performance of RFB against a reference *S. aureus* strain (MSSA), in both free and liposomal forms. In this study, we delve deeper into the potential of RFB, by evaluating its *in vitro* antimicrobial activity against both planktonic and biofilm forms of *S. aureus* clinical isolates recovered from invasive Staphylococcal infections.

## 2 Materials and methods

### 2.1 Reagents

Vancomycin (VAN) was obtained from Sigma-Aldrich (St. Louis, MO, United States) and Rifabutin (RFB) from Pharmacy Biotech AB (Uppsala, Sweden). Thiazolyl Blue Tetrazolium Bromide (MTT) and Crystal violet (CV) were purchased from Panreac Applichem, ITW Reagents (Darmstadt, Germany). Culture media Mueller-Hinton Agar (MHA), Mueller-Hinton Broth (MHB), and Tryptic Soy Broth (TSB) were obtained from Biokar (Pantin, France) and Columbia Agar +5% sheep blood (CA) from BioMérieux (Marcy L’Ètoile, France). All the remaining chemicals used were of analytical grade.

### 2.2 Bacterial isolates

A collection of 114 clinical isolates recovered from invasive Staphylococcal disease, was kindly provided by the “Laboratório de Microbiologia do Serviço de Patologia Clínica do Centro Hospitalar Universitário de Lisboa Central, Lisboa, Portugal.” The pathology laboratory was requested to recover all cases of invasive *S. aureus* isolates recovered during the period from April 14 to September 13, 2021. A case of invasive disease was defined as an isolate of *S. aureus* recovered from blood. All strains were identified as *S. aureus* by Matrix Assisted Laser Desorption Ionization Time-of-Flight (MALDI-TOF) though VITEK® MS system version 3.2 (Biomerieux, Portugal). A methicillin susceptible *S. aureus* ATCC®25923™ (MSSA) was also included in this study as a reference strain, obtained from the American Type Culture Collection (ATCC; Manassas, VA, United States). Bacterial stocks were prepared from overnight cultures on MHA or CA at 37°C and stored in MHB with 20% of glycerol at −80°C. Among the isolates collection, a total of 21 methicillin-resistant strains (MRSA) and 93 methicillin-susceptible strains (MSSA) were recovered.

### 2.3 Bacterial growth evaluation

All isolates were evaluated in terms of growth profiles. For this, bacterial suspensions of each isolate were prepared, from an overnight agar culture, in MHB at 0.5 McFarland turbidity standard, equivalent to 10^8^ colony forming unit per mL (CFU/mL) by measuring optical density (OD) at 600 nm. The bacterial suspensions were incubated at 37°C during 24 h. The OD at 600 nm of each suspension was measured at the following incubation time points: 1.0, 1.5, 2.0, 2.5, 3.0, 3.5, 4.0, 4.5, 5.0, 5.5, 6.0, and 24 h.

### 2.4 Minimum inhibitory concentration determination

The MIC of RFB and VAN was determined for all clinical isolates, by the broth microdilution method, according to the Clinical and Laboratory Standards Institute guidelines (CLSI, 2020), followed by turbidity evaluation. This concentration range was selected based on a preliminary screen to appropriately define the concentration range (data not shown). The antibiotics were diluted in MHB to produce a 2-fold dilution with concentrations ranging from: 0.0002 to 25.0000 μg/mL and 0.02340 to 24.0000 μg/mL for RFB and VAN, respectively. Bacterial suspensions were performed from overnight cultures on MHA diluted in MHB until reaching a value of 0.5 in a McFarland scale. Bacterial suspensions of each isolate were placed in 96-well culture plate at 5 × 10^5^ CFU/mL and incubated with the respective, antibiotic at different concentrations, at 37°C during 24 h, under static conditions. A negative control containing a suspension of each isolate in MHB, without antibiotic, and a sterile control containing only MHB, were performed in parallel. Minimum inhibitory concentrations (MIC) were determined spectrophotometrically, at 570 nm in an iMark™ microplate absorbance reader (Bio-Rad laboratories, Inc., Hercules, CA, United States) and defined as the lowest antibiotic concentration able to prevent visible bacterial growth, resulting in the absence of turbidity. The MIC of the reference strain was assessed once a week as a control of the assay.

### 2.5 Biofilm assembly evaluation

A subset of 40 isolates was selected for evaluation of biofilm formation after initial MIC screening for RFB and VAN on all clinical isolates. This subset included all methicillin-resistant strains (MRSA, *n* = 21), all strains with VCM MIC values of 1.5 μg/mL (*n* = 13), four randomly selected strains with VCM MIC values of 0.750 μg/mL (*n* = 4) and strains with RFB MIC values exceeding 0.025 μg/mL (*n* = 2). This assay was performed as described previously by Stepanovic et al. (2000) with small modifications. Briefly, bacterial suspensions were inoculated with a final concentration of 10^6^ CFU/mL in TSB supplemented with 0.25% of glucose (TSB 0.25%), in 96-well culture plate. Plates were incubated at 37°C for different time points (6, 24, and 48 h), under static conditions. TSB 0.25% without bacterial suspension was used as a sterile control. Biofilm assembly was evaluated by the crystal violet staining method (CV) (Pontes et al., 2016; Ferreira et al., 2017, 2021c). Following each time point, the content of the wells was washed twice with a sterile solution of phosphate buffered saline (PBS) to remove non-adherent bacteria. The attached bacterial cells were air-dried at room temperature (RT) for 15 min and subsequently stained with 200 μL of a CV solution 0.125% (w/v in water) and incubated at RT for further 15 min. Biofilms were washed twice with sterile PBS to remove excess dye, followed by a drying step at RT. Stained biofilms were then dissolved in 200 μL of ethanol and diluted at a ratio of 1:10. The OD was measured at 570 nm using an iMark™ microplate absorbance reader (Bio-Rad laboratories, Inc., Hercules, CA, United States).

### 2.6 Minimum biofilm inhibitory concentration determination

The minimum biofilm inhibitory concentration of 50% bacterial growth (MBIC_50_) in the set of biofilm-forming isolates previous assessed, was subsequently determined for the two evaluated antibiotics. Thus, overnight cultures were inoculated at 10^6^ CFU/mL in 96-well culture plates in TSB 0.25%. Biofilms were assembled under static condition for 24 h at 37°C. Then each well was washed twice with sterile PBS solution. Serial dilutions of the antibiotics, ranging from 0.0002 to 25.0000 μg/mL and 6.25 to 200.00 μg/mL to RFB and VAN, respectively, were performed in TSB 0.25% and added to the respective wells. Plates were incubated for 24 h at 37°C. A negative control containing a suspension of each isolate in TSB 0.25%, without antibiotic, and a sterile control containing only TSB 0.25%, were performed in parallel. The bacterial cell viability was measured by the MTT reduction assay (Ferreira et al., 2021c). The assay was executed as previously described by Brambilla et al. (2017) with some exceptions. After biofilm rinsing with sterile PBS solution, 200 μL of a 125 μg/mL MTT solution in PBS was added and incubated at 37°C during 2 h. Then the MTT solution was removed and replaced by dimethyl sulfoxide (DMSO) to dissolve the MTT formazan product. The OD of the wells was measured at 570 nm using an iMarkTM microplate absorbance reader (Bio-Rad laboratories, Inc., Hercules, CA, United States). MBIC_50_ was defined as the lowest antibiotic concentration able to inhibit more than 50% of bacterial growth compared to untreated controls. The determination of MBIC_50_ was performed by sigmoidal fitting analysis considering a confidence level of 95%. The MBIC_50_ of the reference strain was assessed once a week as a control of the assay.

## 3 Results and discussion

### 3.1 Isolate collection

*Staphylococcus aureus* stands out as one of the most significant pathogens worldwide, particularly due to the clinical and epidemiological relevance of invasive infections caused by MRSA, exhibiting high rates of morbidity and mortality (van Hal et al., 2012; Hassoun et al., 2017). The prevalence of systemic MRSA infections worldwide ranges from 20 to 50 per 100,000 population annually in countries with low and high incidence rates, respectively (van Hal et al., 2012; Hassoun et al., 2017). While there has been a decline in the incidence of MRSA bacteremia over the past decade, it continues to be associated with poorer clinical outcomes compared to infections caused by methicillin susceptible *S. aureus* (MSSA) (Hassoun et al., 2017).

**Figure 1:**
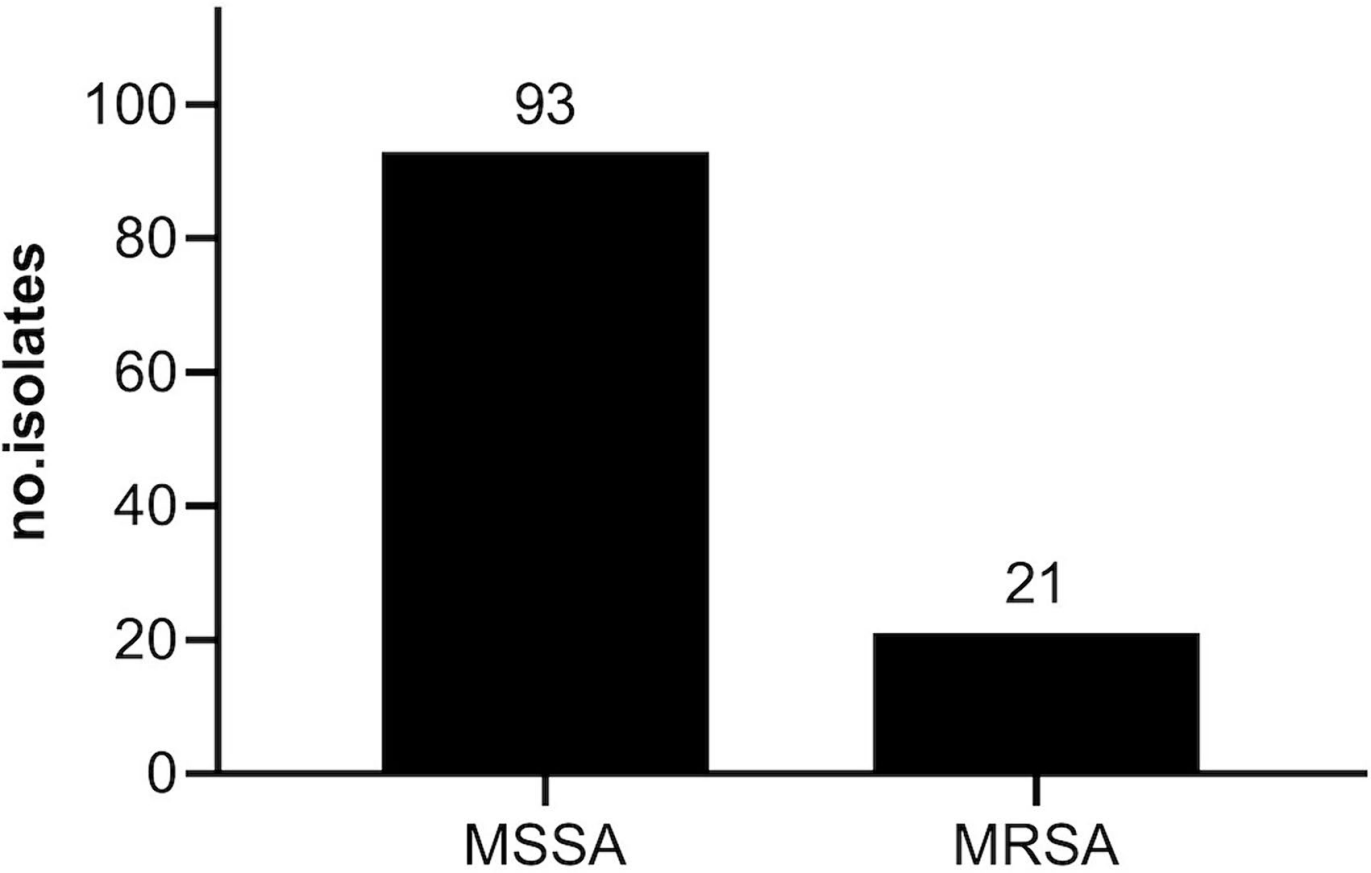
Prevalence of MSSA and MRSA clinical isolates in the study population. Isolates were recovered from invasive infections between April 14 to September 13 of 2021, by the “Laboratório de Microbiologia do Serviço de Patologia Clínica do Centro Hospitalar Universitário de Lisboa Central, Lisboa, Portugal”.

To comprehensively evaluate the antibacterial potential of RFB against *S. aureus* originating from *de facto* infections, a collection of 114 staphylococcal clinical isolates were recovered from bloodstream infections. Among these, 93 (81.58%) isolates were susceptible to methicillin (MSSA) and 21 (18.42%) were methicillin resistant strains (MRSA) (Figure 1). Although, the current work was not designed to assess MRSA prevalence in the Portuguese population, the prevalence of MRSA in our study was consistent with the data from Gagliotti et al. (2021), who reported that, based on the European Antimicrobial Resistance Surveillance Network (EARS-Net), 16.3% of the *S. aureus* strains isolated from bloodstream infections in 2018 were MRSA. Yet, the rate was below the reported from the antimicrobial resistance surveillance report in Europe from 2022, elaborated by the European Center for Disease Prevention and Control (ECDC), which revealed that in 2020, 29.7% of invasive *S. aureus* isolates tested were MRSA in Portugal (WHO and ECDC, 2022).

### 3.2 Planktonic clinical isolates susceptibility to antibiotics

Currently, the glycopeptide, VAN, remains the antibiotic of choice for treatment of MRSA infections. However, strains resistant to or with reduced susceptibility to VAN have been emerging (Shajari et al., 2017). Consequently, VAN is normally administered in combination with other antibiotics, such as rifampicin, to improve the therapeutic efficacy and reduce the emergence of resistant strains. Yet, it has severe adverse side effects associated, due to its toxicity profile. To overcome these drawbacks, studies have demonstrated the advantages of using RFB instead of rifampicin in Staphylococcal infections (Doub et al., 2020; Karau et al., 2020; Ferreira et al., 2021c; Thill et al., 2022).

To determine the susceptibility profile to VAN and RFB, of *S. aureus* clinical isolates recovered from invasive infections, the MIC values were determined. As shown in Table 1, all tested isolates were susceptible to VAN, presenting MIC values ranging from 0.375 to 1.500 μg/mL. The MIC_50_ value, which is the concentration at which 50% of the clinical isolates are inhibited (Schwarz et al., 2010) was 0.750 μg/mL for both MSSA and MRSA strains, constituting 80.6% of MSSA (*n* = 75) and 90.5% of MRSA strains (*n* = 19).

**Table 1:** Distribution of *Staphylococcus aureus* clinical isolates (number of strains) according to VAN MIC value obtained.

MIC^a^ VAN (μg/mL)	Number of strains (%)	
	MSSA (*n* = 93)	MRSA (*n* = 21)
0.375	5 (5.4%)	2 (9.5%)
0.750	75 (80.6%)	19 (90.5%)
1.500	13 (14.0%)	0 (0.0%)

a The lowest antibiotic concentration able to prevent visible growth after 24 h of incubation.

VAN susceptible ≤ 2 μg/mL; VAN intermediate-resistant MIC = 4–8 μg/mL; VAN-resistant MIC ≥ 16 μg/mL; Results are average of technical triplicates.

Rifabutin MIC values (Table 2) ranged from 0.002 to 6.250 μg/mL, with *n* = 43 (46.2%) and *n* = 15 (71.4%) of the MSSA and MRSA strains presenting MIC values of 0.013 μg/mL, consistent with the RFB MIC_50_ value. To the best of our knowledge, there are currently no established breakpoints for RFB against *S. aureus*. Notwithstanding, the data obtained in this study revealed low inhibitory concentrations for RFB for both, planktonic MSSA and MRSA strains and are in line with the previous study from Albano and collaborators who reported a MIC_50_ = 0.016 μg/mL for *S. aureus* strains recovered from periprosthetic joint infections (Albano et al., 2019).

**Table 2:** Distribution of *Staphylococcus aureus* clinical isolates (number of strains) according to RFB MIC value obtained.

RFB MIC^a^ (μg/mL)	Number of strains (%)	
	MSSA (*n* = 93)	MRSA (*n* = 21)
0.002	1 (1.1%)	0 (0.0%)
0.003	6 (6.5%)	0 (0.0%)
0.006	36 (38.7%)	4 (19.0%)
0.013	43 (46.2%)	15 (71.4%)
0.025	5 (5.4%)	2 (9.5%)
0.391	1 (1.1%)	0 (0.0%)
6.250	1 (1.1%)	0 (0.0%)

a The lowest antibiotic concentration able to prevent visible growth after 24 h of incubation.

Results are average of technical triplicates.

Similar susceptibility profiles were obtained for the reference *S. aureus* strain ATCC®25923™ (MSSA), which was tested in parallel. The reference *S. aureus* strain presented MICs of 1.500 and 0.006 μg/mL for VAN and RFB, respectively.

### 3.3 Biofilm clinical isolates susceptibility to antibiotic

It has been known that bacteria organized in biofilms are resistant to most antibiotics, reaching a resistance 1,000 times higher than their planktonic counterparts (Divakar et al., 2019; Doub et al., 2020; Ferreira et al., 2021a; Peng et al., 2022) and researchers believe that more than 80% of chronic infections are associated to bacterial biofilms (Divakar et al., 2019).

In light of the aforementioned considerations, the present study aimed to validate the anti-biofilm activity of RFB in parallel to VAN. For this purpose, a subset of 40 clinical isolates was selected based on the resistance profile previously obtained, with the objective of determining the MBIC_50_ of RFB and VAN through the evaluation of bacterial cell viability. All MRSA clinical isolates, strains exhibiting VAN MIC values of 1.500 μg/mL, four randomly selected strains with VAN MIC of 0.750 μg/mL, and the two strains with the highest RFB MIC values (RFB MIC = 6.250 and 0.391 μg/mL) were included in the sub-analysis.

The ability of the 40 selected isolates to form biofilms structures was initially confirmed, with mature biofilms observed after 24 h of incubation for all isolates (data not shown). The 24 h old biofilm susceptibility to VAN ranged from 10.00 to more than 200.00 μg/mL, while for the RFB from 0.005 to more than 25.000 μg/mL (Tables 3, 4, respectively). As expected, all clinical isolates in this subset displayed higher inhibitory concentration to VAN in biofilm compared to planktonic state (Tables 1, 3). Indeed, MBIC_50_ superior to 200.00 μg/mL was observed for 47.4% (*n* = 9) and 23.8% (*n* = 5) of the selected MSSA and MRSA strains, respectively. For the reference strain ATCC®25923™, a MBIC_50_ higher than 200.00 μg/mL was achieved, exhibiting a biofilm susceptibility profile similar to the one observed for the selected set of clinical isolates.

**Table 3:** Distribution of *Staphylococcus aureus* clinical isolates (number of strains) according to VAN MBIC_50_ values obtained.

VAN MBIC_50_^a^ (μg/mL)	Number of strains (%)	
	MSSA (*n* = 19)	MRSA (*n* = 21)
10 to <20	1 (5.3%)	2 (9.5%)
20 to <30	3 (15.8%)	2 (9.5%)
30 to <40	2 (10.5%)	2 (9.5%)
40 to <50	3 (15.8%)	0 (0.0%)
50 to <6 0	1 (5.3%)	3 (14.3%)
60 to <70	0 (0.0%)	0 (0.0%)
70 to <80	0 (0.0%)	2 (9.5%)
80 to <90	0 (0.0%)	1 (4.8%)
90 to <100	0 (0.0%)	1 (4.8%)
100 to <150	0 (0.0%)	2 (9.5%)
150 to <200	0 (0.0%)	1 (4.8%)
>200	9 (47.4%)	5 (23.8%)

a The lowest antibiotic concentration able to inhibit more than 50% of biofilm growth after 24 h of incubation, determined by MTT assay.

Results are average of technical triplicates.

**Table 4:** Distribution of *Staphylococcus aureus* clinical isolates (number of strains) according to RFB MBIC_50_ values obtained.

RFB MBIC_50_^a^ (μg/mL)	Number of strains (%)	
	MSSA (*n* = 19)	MRSA (*n* = 21)
0.005 to <0.010	1 (5.3%)	2 (9.5%)
0.010 to <0.050	9 (47.4%)	13 (61.9%)
0.050 to <0.100	4 (21.1%)	1 (4.8%)
0.100 to <0.500	2 (10.5%)	1 (4.8%)
12.500 to <1.000	0 (0.0%)	0 (0.0%)
1.000 to <25.000	2 (10.5%)	1 (4.8%)
>25.000	1 (5.3%)	3 (14.3%)

a The lowest antibiotic concentration able to inhibit more than 50% of biofilm growth after 24 h of incubation, determined by MTT assay.

Results are average of technical triplicates.

Previous studies corroborate this increased resistance in bacteria organized in biofilms compared with their planktonic counterparts. A study conducted by Rose and Poppens (2009) demonstrated an 8-fold increase in VAN anti-biofilm activity in relation to VAN MIC values in a collection of 40 MRSA clinical isolates. In more recent publications, MICs values for VAN lower than 4 μg/mL were found in a set of clinical isolates recovered from healthy, subclinical and clinical mastitic human milk samples from lactating women, while in the same isolates organized in biofilms, treatment with VAN at 4x MIC led to significant increase in biofilm biomass compared with the untreated control (Angelopoulou et al., 2020). Furthermore, Douthit et al. (2020) suggested that VAN can effectively eradicate *S. aureus* biofilms, but only in concentrations above 6,000 μg/mL.

The majority of MSSA (*n* = 9; 47.4%) and MRSA (*n* = 13; 61.9%) isolates presented a decrease of bacterial cell viability above 50% for RFB concentrations ranging from 0.010 to 0.050 μg/mL. Surprisingly, one MSSA (5.3%) and three (14.3%) MRSA strains did not show a biofilm decrease above 50% with the tested concentrations, leading to the conclusion that these strains presented RFB MBIC_50_ higher than 25.000 μg/mL. As observed for some clinical isolates, the reference strain showed RFB MBIC_50_ values similar to the respective MIC (RFB MIC ATCC®25923™ = 0.006 μg/mL; RFB MBIC_50_ ATCC®25923™ = 0.005 μg/mL).

To the best of our knowledge, the *in vitro* anti-biofilm activity of RFB have not yet been assessed in *S. aureus* clinical isolates recovered from bloodstream infections. However, previous studies have already demonstrated the antibacterial potential of RFB in the treatment of *S. aureus* associated to biofilm infections. Indeed, Doub et al. (2020) validated the *in vivo* benefit of RFB instead of rifampicin in patients with chronic staphylococcal infections associated to prosthetic material. Remarkably, none of the patients experienced recurrence of staphylococcal infections or reported any adverse side effects with RFB therapy (Doub et al., 2020). Moreover, Karau et al. (2020) conducted a study investigating the therapeutic efficacy of RFB either alone or in combination with VAN in a rat model of foreign body osteomyelitis. Their results demonstrated a higher reduction in colony forming units (CFU) counts in the animals treated with RFB in combination with VAN than the common treatment, rifampicin with VAN, suggesting once more the therapeutic potential of rifabutin in these type of infections (Karau et al., 2020).

## 4 Conclusion

The increase emergency of multidrug-resistant strains, as well as the poor biofilm penetration capacity of available antibiotics used for the treatment of infections caused by *S. aureus*, prompt the research of novel therapeutic approaches. However, the discovery and development of new antibiotics face significant challenges and non-antibiotic therapeutic strategies have not yet proven to be a viable solution. In contrast, RFB, as a repurposing antibiotic, has been gaining prominence as demonstrated in several studies. To further validate the potential of RFB as a strong therapeutic option for *S. aureus* invasive infections, we evaluated the antibacterial activity of RFB in a collection of 114 *S. aureus* clinical isolates recovered from invasive staphylococcal infections. RFB demonstrated to be a promising antibiotic against *S. aureus* not only in planktonic state but also in biofilm state with MBIC_50_ values similar to the respective MICs. Importantly, no significant differences in terms of antibacterial effect against MSSA or MRSA strains under study were observed. These are promising results that suggests that RFB may be a useful option, especially in biofilm-associated infections. Yet, further clinical investigations are warranted to confirm rifabutin efficacy against *S. aureus* in comparison to standard treatments and to define the optimal dosing, treatment duration or even combination therapy.

## Data Availability

The original contributions presented in the study are included in the article/Supplementary material, further inquiries can be directed to the corresponding author.
