# Supplementary material for: Rifabutin: a repurposed antibiotic with high potential against planktonic and biofilm staphylococcal clinical isolates
Source: Front Microbiol. 2024 Oct 10;15:1475124. doi: 10.3389/fmicb.2024.1475124 (PMC11499150; doi:10.3389/fmicb.2024.1475124)
Supplement: Supplementary file 1 [file Table_1.DOCX]

Supplementary Material

# Supplementary Data

TABLE S1 Clinical isolates collection – MIC and MBIC_50_ obtained values.

| ID | Strain | MIC VCM (μg/mL) | MIC RFB (μg/ml) | MBIC_50_ VCM (μg/mL) | MBIC_50_ RFB (μg/mL) |
| --- | --- | --- | --- | --- | --- |
| SA-1 | MSSA | 1.500 | 0.006 | 43.430 | 0.013 |
| SA-2 | MSSA | 0.750 | 0.003 | nd | nd |
| SA-3 | MSSA | 0.750 | 0.003 | nd | nd |
| SA-4 | MSSA | 1.500 | 0.006 | 17.920 | 0.067 |
| SA-5 | MSSA | 0.750 | 0.006 | nd | nd |
| SA-6 | MSSA | 1.500 | 0.006 | >200.000 | 0.017 |
| SA-7 | MSSA | 0.750 | 6.250 | 26.940 | 20.39 |
| SA-8 | MSSA | 0.750 | 0.003 | nd | nd |
| SA-9 | MSSA | 0.750 | 0.003 | nd | nd |
| SA-10 | MSSA | 0.750 | 0.013 | nd | nd |
| SA-11 | MSSA | 0.375 | 0.006 | nd | nd |
| SA-12 | MSSA | 0.750 | 0.006 | nd | nd |
| SA-13 | MRSA | 0.375 | 0.006 | 36.660 | 0.023 |
| SA-14 | MRSA | 0.750 | 0.025 | >200.000 | 0.034 |
| SA-15 | MSSA | 0.750 | 0.013 | nd | nd |
| SA-16 | MSSA | 1.500 | 0.006 | >200.000 | 0.032 |
| SA-17 | MSSA | 0.750 | 0.025 | nd | nd |
| SA-18 | MSSA | 0.750 | 0.006 | nd | nd |
| SA-19 | MSSA | 0.750 | 0.002 | nd | nd |
| SA-20 | MRSA | 0.750 | 0.013 | 82.500 | 0.018 |
| SA-21 | MSSA | 0.750 | 0.006 | nd | nd |
| SA-22 | MSSA | 0.750 | 0.025 | 27.350 | 0.069 |
| SA-23 | MRSA | 0.750 | 0.006 | 54.180 | 0.029 |
| SA-24 | MSSA | 0.750 | 0.006 | nd | nd |
| SA-25 | MSSA | 0.750 | 0.006 | nd | nd |
| SA-26 | MSSA | 0.750 | 0.003 | nd | nd |
| SA-28 | MSSA | 0.750 | 0.006 | nd | nd |
| SA-29 | MSSA | 0.375 | 0.006 | nd | nd |
| SA-30 | MSSA | 0.750 | 0.006 | nd | nd |
| SA-31 | MSSA | 0.750 | 0.006 | nd | nd |
| SA-32 | MSSA | 0.750 | 0.013 | >200.000 | 0.011 |
| SA-33 | MSSA | 0.375 | 0.013 | nd | nd |
| SA-34 | MSSA | 0.750 | 0.013 | nd | nd |
| SA-35 | MRSA | 0.750 | 0.013 | 54.710 | 0.037 |
| SA-36 | MRSA | 0.750 | 0.006 | 55.110 | 0.006 |
| SA-37 | MSSA | 0.750 | 0.013 | nd | nd |
| SA-38 | MSSA | 0.750 | 0.013 | nd | nd |
| SA-39 | MSSA | 0.750 | 0.013 | nd | nd |
| SA-40 | MSSA | 0.750 | 0.013 | nd | nd |
| SA-41 | MSSA | 0.750 | 0.013 | nd | nd |
| SA-44 | MRSA | 0.750 | 0.025 | 195.100 | 0.063 |
| SA-45 | MSSA | 0.750 | 0.006 | nd | nd |
| SA-46 | MSSA | 0.750 | 0.006 | nd | nd |
| SA-47 | MSSA | 0.750 | 0.003 | nd | nd |
| SA-48 | MSSA | 0.375 | 0.013 | nd | nd |
| SA-49 | MSSA | 0.750 | 0.013 | 48.090 | 0.034 |
| SA-50 | MSSA | 0.750 | 0.013 | nd | nd |
| SA-51 | MSSA | 0.750 | 0.013 | nd | nd |
| SA-52 | MSSA | 0.750 | 0.391 | >200.000 | 10.8 |
| SA-53 | MSSA | 0.750 | 0.013 | nd | nd |
| SA-54 | MSSA | 0.750 | 0.006 | nd | nd |
| SA-55 | MSSA | 0.750 | 0.013 | nd | nd |
| SA-56 | MSSA | 0.750 | 0.013 | nd | nd |
| SA-57 | MSSA | 0.375 | 0.006 | nd | nd |
| SA-58 | MRSA | 0.750 | 0.013 | 12.820 | 5.363 |
| SA-59 | MSSA | 0.750 | 0.006 | nd | nd |
| SA-60 | MSSA | 0.750 | 0.025 | nd | nd |
| SA-61 | MSSA | 0.750 | 0.006 | nd | nd |
| SA-62 | MRSA | 0.750 | 0.013 | >200.000 | 0.007 |
| SA-63 | MSSA | 1.500 | 0.013 | 54.350 | >25 |
| SA-64 | MSSA | 0.750 | 0.006 | nd | nd |
| SA-65 | MSSA | 0.750 | 0.006 | nd | nd |
| SA-66 | MSSA | 0.750 | 0.013 | nd | nd |
| SA-67 | MRSA | 0.750 | 0.006 | 34.310 | >25 |
| SA-68 | MSSA | 0.750 | 0.006 | nd | nd |
| SA-69 | MRSA | 0.750 | 0.013 | >200 | 0.02 |
| SA-70 | MSSA | 0.750 | 0.006 | nd | nd |
| SA-71 | MSSA | 1.500 | 0.013 | >200.000 | 0.025 |
| SA-72 | MSSA | 1.500 | 0.006 | 23.880 | 0.042 |
| SA-73 | MSSA | 0.750 | 0.013 | nd | nd |
| SA-74 | MSSA | 0.750 | 0.013 | nd | nd |
| SA-75 | MSSA | 1.500 | 0.006 | >200.000 | 0.006 |
| SA-76 | MSSA | 0.750 | 0.013 | nd | nd |
| SA-77 | MSSA | 0.750 | 0.006 | nd | nd |
| SA-78 | MSSA | 1.500 | 0.013 | >200.000 | 0.269 |
| SA-79 | MSSA | 0.750 | 0.013 | nd | nd |
| SA-80 | MSSA | 0.750 | 0.006 | nd | nd |
| SA-81 | MSSA | 0.750 | 0.013 | nd | nd |
| SA-82 | MSSA | 1.500 | 0.013 | 31.530 | 0.446 |
| SA-83 | MSSA | 0.750 | 0.006 | nd | nd |
| SA-84 | MRSA | 0.750 | 0.013 | >200.000 | 0.018 |
| SA-85 | MSSA | 0.750 | 0.013 | nd | nd |
| SA-86 | MSSA | 0.750 | 0.013 | nd | nd |
| SA-87 | MSSA | 0.750 | 0.006 | nd | nd |
| SA-88 | MSSA | 0.750 | 0.013 | 31.530 | 0.014 |
| SA-89 | MRSA | 0.750 | 0.013 | 21.880 | 0.049 |
| SA-90 | MRSA | 0.750 | 0.013 | 75.130 | 0.045 |
| SA-91 | MRSA | 0.750 | 0.013 | 20.130 | >25 |
| SA-92 | MSSA | 0.750 | 0.025 | nd | nd |
| SA-93 | MSSA | 1.500 | 0.013 | >200.000 | 0.071 |
| SA-94 | MSSA | 1.500 | 0.013 | 41.850 | 0.036 |
| SA-95 | MSSA | 0.750 | 0.013 | nd | nd |
| SA-96 | MSSA | 0.750 | 0.013 | nd | nd |
| SA-97 | MSSA | 0.750 | 0.013 | nd | nd |
| SA-98 | MSSA | 0.750 | 0.013 | nd | nd |
| SA-99 | MRSA | 0.750 | 0.013 | >200.000 | 0.014 |
| SA-100 | MSSA | 0.750 | 0.013 | nd | nd |
| SA-101 | MSSA | 1.500 | 0.006 | >200.000 | 0.095 |
| SA-102 | MSSA | 0.750 | 0.006 | nd | nd |
| SA-103 | MSSA | 0.750 | 0.013 | nd | nd |
| SA-104 | MRSA | 0.750 | 0.013 | 18.400 | >25 |
| SA-105 | MSSA | 0.750 | 0.006 | nd | nd |
| SA-106 | MSSA | 0.750 | 0.006 | nd | nd |
| SA-107 | MSSA | 0.750 | 0.013 | nd | nd |
| SA-108 | MSSA | 0.750 | 0.013 | nd | nd |
| SA-109 | MSSA | 0.750 | 0.013 | nd | nd |
| SA-110 | MSSA | 0.750 | 0.006 | nd | nd |
| SA-111 | MSSA | 0.750 | 0.025 | nd | nd |
| SA-112 | MRSA | 0.750 | 0.013 | 136.900 | 0.022 |
| SA-113 | MSSA | 0.750 | 0.013 | nd | nd |
| SA-114 | MRSA | 0.375 | 0.013 | 92.810 | 0.101 |
| SA-115 | MRSA | 0.750 | 0.013 | 117.000 | 0.012 |
| SA-116 | MRSA | 0.750 | 0.013 | 72.360 | 0.043 |
| SA-117 | MSSA | 0.750 | 0.013 | nd | nd |

nd – not determined
